# Genetic Insights Into ADHD Biology

**DOI:** 10.3389/fpsyt.2018.00251

**Published:** 2018-06-07

**Authors:** Victoria Hayman, Thomas V. Fernandez

**Affiliations:** ^1^Physiology Department, McGill University, Montreal, QC, Canada; ^2^Child Study Center, Department of Psychiatry, Yale University School of Medicine, New Haven, CT, United States

**Keywords:** attention-deficit/hyperactivity disorder, GWAS (genome-wide association study), copy number variation (CNV), candidate gene association, network analysis, pathway analysis, expression profiling, cerebellum

## Abstract

ADHD is a neurobiological disorder with a large worldwide prevalence causing significant impairment in children, adolescents, and adults. While there is general agreement about genetic contributions toward the disorder, progress in leveraging genetics to learn more about the biology and risk factors for ADHD has been limited. In this perspective, we identified 105 genes from the literature showing at least nominal statistical significance in association with ADHD. We analyzed these genes for enrichment in biological pathways and in known interacting biological networks. We also analyzed the expression patterns of candidate genes across brain regions and across periods of human development. From our analysis, we identify 14 genes that cluster within an interactive gene network, with enrichment in nitric oxide synthase and alpha-1 adrenergic pathways. Furthermore, these genes show enrichment for expression in the cerebellum during childhood through young adulthood, and in the cortex in adolescence and young adulthood. Gene discovery holds great potential for elucidating the unknown biological underpinnings of ADHD. Genome-wide sequencing efforts are underway and are likely to provide important insights that can be leveraged for new treatments and interventions.

## Introduction

Attention-deficit/hyperactivity disorder (ADHD) is the most common neurodevelopmental disorder in childhood and is characterized by persistent and developmentally inappropriate hyperactivity, impulsivity and inattention (1). There are three subtypes of ADHD, categorized by type of behavioral symptoms: predominantly hyperactive/impulsive, predominantly inattentive, and combined. The disorder can affect children, adolescents, and adults, yet symptoms begin in childhood. The worldwide prevalence among children and adolescents is estimated to be about 5% (2), and it is estimated that 65% of affected individuals continue to exhibit ADHD symptoms into adulthood (3). ADHD prevalence in adults is estimated to be 2.5% (4). In childhood, ADHD is more prevalent in males than females, with a gender ratio of 3:1 (5). In adults, this ratio disparity narrows considerably, with a range between 1:1 and 2:1 (6). The disorder often coexists with other psychiatric conditions, the most common of which include mood, anxiety, conduct and oppositional defiant disorders (7).

ADHD etiology is believed to be multifactorial. It is known that genetics plays a significant role in the disorder; twin studies indicate the heritability value of ADHD to be 0.76 (8). In addition, environmental risk factors of ADHD have been proposed. These include environmental contaminants such as lead and polychlorinated biphenyls (9), as well as biological indices such as extremely low birth weight (10). Prenatal exposure to nicotine (11), stress (12), and alcohol (13) have also been suggested as increasing ADHD risk in children.

This perspective will begin with a brief overview of treatment and structural neuroimaging findings, then will amalgamate and succinctly outline findings from genetic research studies of ADHD thus far. We will then incorporate these findings into a new analysis showing enrichment of ADHD genetic variants in particular biological pathways and networks. We advocate for continued updating of such analyses in the future, as more high confidence genetic risk variants are uncovered. These analyses will elucidate essential spatial, temporal, and biological network characteristics of ADHD. This knowledge is a prerequisite for advancing treatment and prevention efforts.

## Treatment

Best Practice recommendations for ADHD treatment generally depend on the age of the individual. For preschool-aged children, cognitive behavioral therapy (CBT) is recommended as a first-line treatment, while for children aged 6 years and above, pharmacological treatment is advised (14). Commonly prescribed medications to treat ADHD include stimulants, which are generally subdivided into the classes of methylphenidates (e.g., Ritalin, Concerta) and amphetamines (e.g., Adderall, Vyvanse). Studies comparing the two classes of stimulants have produced mixed results, and there is no overwhelming evidence suggesting that one class is superior to the other (15). Non-stimulant options are also available and are usually prescribed when a patient does not respond to stimulants or does not tolerate them (e.g., due to increase in tic frequency, insomnia, or loss of appetite). Non-stimulant medications include atomoxetine, guanfacine, and clonidine. Evidence for the efficacy of these non-stimulant medications in the treatment of ADHD symptoms is ample, yet not as robust as evidence for stimulants (14). In 1999, a major study found that combination (pharmacological + behavioral) therapy and medication-only therapy were both superior to behavioral-only therapy and routine community care for reducing ADHD symptoms (16). However, follow-up studies indicate that this finding may not be conclusive (17–19).

## Structural neuroimaging

Most MRI studies of ADHD have been conducted on males, most likely due to the gender disparity of the disorder. There is evidence that the ADHD brain has a smaller global volume (20) and an overall decrease in white matter volume (21) compared to controls. Additionally, specific brain regions have been associated with morphological changes. One such area includes the fronto-striatal structures (lateral prefrontal cortex, dorsal anterior cingulate cortex, and dorsal striatum) (22), which are involved in cognitive tasks, control of movement, and response inhibitions (23, 24). Specifically, the caudate nucleus and globus pallidus, which together form part of the basal ganglia, have been found to be smaller in children and adolescents with ADHD (25). Reduced gray matter volume in the dorsolateral prefrontal cortex has also been observed, with no difference upon comparison of the various ADHD subtypes (20, 26). More recently, the cerebellum, long thought to be solely responsible for motor control, has been found to affect cognition (27). Reduced cerebellar vermis area and volume have been associated with ADHD in both males and females (28–30), and poorer clinical outcomes have been associated with decreased volumes of right and left inferior-posterior lobes of the cerebellum in childhood through adolescence (29). Furthermore, postural deficits have been reported in ADHD, leading to hypotheses of cerebellar involvement (31–33), and possibly playing a role in the increased risk of injury in school-age children with ADHD (34).

## Genome-wide association studies

Genome-wide association studies (GWAS) are hypothesis-free analyses that have been used to scan the genomes of children, adolescents and adults in search of genetic markers of ADHD (35). This type of study is performed using a microarray platform to genotype hundreds of thousands to millions of single nucleotide polymorphisms (SNPs), where the particular SNPs are chosen based on linkage disequilibrium distribution within the genome (36). Until recently, no genetic variants in ADHD have achieved genome-wide significance (*p* ≤ 5 × 10^−8^) via GWAS. This is not entirely surprising; given the heterogeneity and multifactorial nature of the disorder, very large cohorts are likely required to achieve this stringent level of significance. In 2017, Demontis et al. performed a meta-analysis of prior GWAS studies, which included 20,183 ADHD cases and 35,191 controls, to identify 12 loci surpassing the threshold of genome-wide significance (37). The majority of prominent genes identified via ADHD GWAS thus far correspond to the largest genes in the human genome (38). Additionally, there has been little overlap among top gene findings and none of these findings have included “classic” ADHD candidate genes (39). Further GWAS investigations with larger cohorts will be required to discover more ADHD risk genes and confirm prior findings.

## Candidate gene association studies

ADHD candidate gene studies, unlike GWAS, select genes to study based on *a priori* hypotheses regarding the function of the genes and their presumed relationship to the disorder (40). While many candidate ADHD genes have been previously assessed using this model, candidate genes studies are no longer frequently pursued due the development of new, broader-reaching genomic tools and greater recognition of the weaknesses of this approach. The predetermined selection of SNPs used in this type of study lowers the probability of identifying a marker associated with ADHD, and many earlier studies did not control for ethnic differences in allele frequencies between cases and controls (35).

## Identifying potential ADHD genes from the literature

Supplementary Table S1 lists 105 ADHD candidate genes identified by reviewing genetic studies of ADHD in children, adults, or both. Two databases—PubMed and Google Scholar—were used to query search terms “ADHD genetics” and “ADHD candidate genes” (accessed July 15, 2017). No cross-disorder studies were included. A gene was included in Supplementary Table S1 if an association with at least one ADHD subtype diagnosis was reported as statistically significant in at least one association study (*p* < 0.05 for GWAS, copy number variant, and candidate gene association studies). It is important to note, however, that these genes did not necessarily achieve genome-wide significance.

## Pathway and network analysis

**Figure 1:**
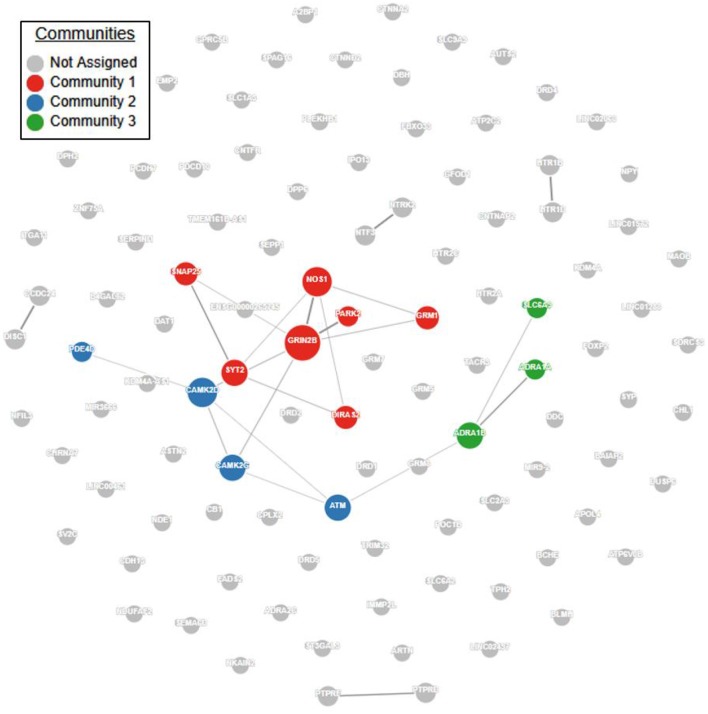
ADHD candidate gene network interaction analysis. Using the GeNets network analysis tool (https://apps.broadinstitute.org/genets), we mapped all 105 ADHD candidate genes obtained from the literature (see Supplementary Table S1) onto the InWeb3 meta-network to determine whether they are functionally connected. The density of the mapped network (density = number of edges/number of possible edges) was greater than 95% of randomly sampled gene sets, indicating that the network is significantly more connected than random (*p* < 2.3E-03). In the network, node (gene) size is proportional to the number of connections. The color is assigned by community, defined as a gene set that is more connected to one another than to another group of genes. Results from this analysis are available in interactive form here: https://www.broadinstitute.org/genets#/computations/5a5d08f9a81b0d6eff609b4b.

Pathway and network analyses that utilize genes of interest may aid in confirming or refuting prior hypotheses regarding genes and biological pathways involved in ADHD and may ultimately provide new insights into the underlying biology of the disorder. First, we performed a protein-protein interaction analysis using GeNets (http://apps.broadinstitute.org/genets, default settings, InWeb3 background meta-network). ADHD genes in Supplementary Table S1 displayed significantly more connectivity than expected by chance (Figure 1, *p* < 2E-03). Furthermore, 14 genes were found to organize within three “communities.” Communities are sets of genes that are more connected to one another than they are to other groups of genes; they can help identify functional modules (Figure 1, Supplementary Table S2). Full results of the GeNets interaction analysis can also be viewed interactively here: https://www.broadinstitute.org/genets#/computations/5a5d08f9a81b0d6eff609b4b.

**Table 1:** Selected ADHD candidate genes based on gene network interaction analysis.

**Community**	**Gene symbol**	**Description**
1	*DIRAS2*	DIRAS family, GTP-binding RAS-like 2
1	*GRIN2B*	glutamate receptor, ionotropic, N-methyl D-aspartate 2B
1	*GRM1*	glutamate receptor, metabotropic 1
1	*NOS1*	nitric oxide synthase 1 (neuronal)
1	*PARK2*	parkin RBR E3 ubiquitin protein ligase
1	*SNAP25*	synaptosomal-associated protein, 25kDa
1	*SYT2*	synaptotagmin II
2	*ATM*	ataxia telangiectasia mutated
2	*CAMK2D*	calcium/calmodulin-dependent protein kinase II delta
2	*CAMK2G*	calcium/calmodulin-dependent protein kinase II gamma
2	*PDE4D*	phosphodiesterase 4D, cAMP-specific
3	*ADRA1A*	adrenoceptor alpha 1A
3	*ADRA1B*	adrenoceptor alpha 1B
3	*SLC6A9*	solute carrier family 6 (neurotransmitter transporter, glycine), member 9

Next, we used all candidate genes in Table 1 to perform an enrichment analysis workflow using MetaCore (http://portal.genego.com/, version 6.33, Thomson Reuters, default settings). Results are shown in Supplementary Table S2. Top enriched pathways include nicotine signaling (FDR adjusted *p* = 7.3E-04) and NMDA receptor trafficking (*p* = 3.3E-05). These are notable findings, with convergent evidence from pharmacology and basic science. For example, bupropion is used off-label in the United States as an alternative to stimulants to treat ADHD; this medication is a non-competitive antagonist of nicotinic acetylcholine receptors (41). Cognitive benefits (improved recognition memory) have been measured in ADHD patients after low dose administration of the non-competitive nicotinic antagonist mecamylamine (42). Furthermore, nicotine is known to act at nicotinic acetylcholine receptors (nAChR) to enhance selective attention (43–45), and mice with a deletion of the alpha-7 nAChR show cognitive deficits including reduced sustained attention (46, 47). Additionally, several studies have reported a higher likelihood of ADHD and related symptoms when an individual is exposed to nicotine in utero, although there is limited evidence to establish causality (48, 49). It is possible that future studies examining gene x environment interactions, querying risk for ADHD after prenatal nicotine exposure among individuals harboring mutations in nicotinic pathway genes, may clarify the relationship. On a related note, evidence is beginning to accumulate regarding a correlation between ADHD and prenatal exposure to cannabis (50–53). Cannabinoid receptor signaling was also a significantly enriched pathway in our analysis (Supplementary Table S2) and may warrant further attention in future studies.

With regard to NMDA signaling, this system has previously been hypothesized to contribute to the pathology of ADHD (54, 55). Additionally, the selective noradrenaline reuptake inhibitor atomoxetine, commonly prescribed for the treatment of ADHD, has been shown to block NMDA-induced membrane currents (56).

A MetaCore analysis of all candidate genes identified the transmission of nerve impulses as the top process network (*p* = 1.9E-14), with localization at the synapse (*p* = 7E-05–1.6E-06) (Supplementary Table S2). This is in agreement with hypotheses about the role of neurotransmission dysregulation in ADHD etiology (54, 57, 58).

We then performed a second enrichment analysis using MetaCore, this time limiting to a set of 14 interactive genes identified by GeNets (Table 1). Results are shown in Supplementary Table S3. This selective gene list showed greatest enrichment for nitric oxide synthase (NOS) signaling (*p* = 5.5 E-04) and alpha-1 adrenergic receptor signaling (*p* = 1E-03). In animal studies, low expression of NOS has been associated with an inattentive phenotype, and methylphenidate administration has been associated with increased NOS expression (59, 60). Furthermore, a NOS inhibiting drug administered to mice modulated their hyperactivity response to methylphenidate (61), and neuronal nitric oxide synthase knockout mice display several ADHD-like behaviors (62). The adrenergic system has long been hypothesized to be involved in ADHD etiology based on the pharmacological response to adrenergic agonists in humans, animal studies showing distractibility and hyperactivity with noradrenergic depletion, and improvement with noradrenergic stimulation (63). Another enriched pathway in this analysis is the “neuroprotective action of lithium” (*p* = 1.1E-03). A downstream action of lithium is to attenuate NMDA receptor activity (64), which reinforces prior hypotheses of NMDA pathway involvement in the etiology of ADHD.

It is worth noting that previous pathway analyses have been reported in ADHD (65–71). Some findings overlap with what we report here: Cristino et al. (69) found that ADHD-associated genes are significantly more interconnected in a protein-protein interaction network than expected by chance; Stergiakouli et al. (66) identified recurrent variants in the nicotinic receptor subunit gene *CHRNA7*; Yang et al. (68) identified enrichments in cell adhesion and glutamate synaptic development; Mooney et al. (71) identified nitric oxide signaling; and all identified at least some pathways involved in central nervous system development. However, at the specific pathway level, there are considerably more differences than similarities between studies. It is not surprising that this is the case, considering the evolving collection of genetic variants that have been identified over the years, the differing methodologies for selecting genes, and differing pathway enrichment analysis tools employed. Our study draws genes from the largest variant pool to date, including the largest meta-analysis of GWAS studies, candidate gene studies, and CNV studies. It is encouraging that we see some overlap with previous pathway studies, that neurodevelopment and function is consistently enriched, and that we are seeing convergence with evidence from pharmacology, neuroimaging, and basic science.

## Findings in relation to the brain

Finally, we performed a specific expression analysis of our ADHD candidate genes (http://genetics.wustl.edu/jdlab/csea-tool-2/) (72, 73). We applied a specificity index statistic (pSI) threshold of 0.05 and determined the periods of human development and brain regions enriched for the expression of our candidate gene lists. When examining all candidate genes listed in Supplementary Table S1, we found significant enrichment for expression in the cerebellum (late fetal), cortex (adolescence, young adulthood), striatum (early mid-fetal, early infancy, early childhood, adolescence, young adulthood), and thalamus (late fetal and adolescence) (Supplementary Table S4). When limiting this analysis to the set of 14 interactive candidate genes in Table 1, we found significant enrichment for expression in the cerebellum (late fetal, early childhood, mid-late childhood, adolescence, young adulthood) and cortex (adolescence, young adulthood) (Supplementary Table S5). The cerebellum has recently been highlighted as important for sustained attention in adolescents (74), and reduced volume and connectivity in the cerebellum has been reported in ADHD (75, 76). Furthermore, several studies have reported atypical functional imaging patterns in prefrontal cortex and cerebellum in ADHD (77, 78). Studies have previously indicated that early markers for the development of ADHD may exist in infants and young children (79, 80). Based on this information and our expression enrichment analyses, further investigations are warranted to determine if ADHD candidate gene expression is enriched at early developmental periods, and the effects of this enrichment on ADHD biology.

## Conclusions

Risk gene identification in ADHD has tremendous potential to teach us about underlying biology, including susceptible periods during human development. In this perspective, we demonstrate this potential using the best available evidence for gene association from the literature. However, these association studies are often underpowered and have largely focused on pre-selected candidate genes of interest, which can potentially bias downstream findings. Further efforts toward genome-wide and statistically robust risk gene identification are crucial for leveraging genetics to learn about biology. These studies are underway in our lab and others, with the hope that the findings will lead to improved treatments and interventions that will improve the lives of those with ADHD.

## Author contributions

Both authors (VH and TF) contributed to the conceptualization, data analysis, and writing of this manuscript.

### Conflict of interest statement

The authors declare that the research was conducted in the absence of any commercial or financial relationships that could be construed as a potential conflict of interest.

## Supplementary material

Supplementary Table S1 **105 ADHD candidate genes**. 105 ADHD candidate genes were identified through a review of GWAS, copy number variant, and candidate gene association studies. A gene is included in this table if an association with at least one ADHD subtype diagnosis was reported as statistically significant (*p* < 0.05) in at least one association study. These genes did not necessarily achieve genome-wide significance.

Supplementary Table S2 **105 ADHD candidate genes pathway and network enrichment analysis**. Results of enrichment analysis for canonical pathway maps and process networks using MetaCore (GeneGo, Inc.). *P*-values and false discovery rates were calculated based on the proportion of network objects that coincide with 105 ADHD candidate genes.

Supplementary Table S3 **14 ADHD candidate genes pathway and network enrichment analysis**. Results of enrichment analysis for canonical pathway maps and process networks using MetaCore (GeneGo, Inc.). *P*-values and false discovery rates were calculated based on the proportion of network objects that coincide with the selected list (after network interaction analysis; see Figure 1, Table 1) of 14 ADHD candidate genes.

Supplementary Table S4 **105 ADHD candidate genes spatiotemporal enrichment analysis**. Enrichment analysis examining overlap between 105 ADHD candidate genes and known expression of these genes in various brain regions across different periods of development. Values given are the Fisher's Exact *p*-values, followed by the Benjamini-Hochberg (BH) corrected values in parentheses. Any result with BH *p*-value less than 0.05 is highlighted.

Supplementary Table S5 **14 ADHD candidate genes spatiotemporal enrichment analysis**. Enrichment analysis examining overlap between 14 ADHD candidate genes (selected list after network interaction analysis; see Figure 1, Table 1) and known expression of these genes in various brain regions across different periods of development. Values given are the Fisher's Exact *p*-values, followed by the Benjamini-Hochberg (BH) corrected values in parentheses. Any result with BH *p*-value less than 0.05 is highlighted.
